# Urogenital schistosomiasis in schoolchildren in the lake zones of Kankossa and Oued Rawdha, southern Mauritania: The first parasitological and malacological survey

**DOI:** 10.1371/journal.pntd.0012505

**Published:** 2024-09-25

**Authors:** Lemat Nakatt, Papa Mouhamadou Gaye, Mohamed Ouldabdallahi Moukah, Binta Niang, Leonardo Basco, Stephane Ranque, Ali Ould Mohamed Salem Boukhary

**Affiliations:** 1 Université de Nouakchott, Faculté des Sciences et Techniques, UR génomes et milieux, Nouakchott, Mauritanie; 2 Aix Marseille Univ, AP-HM, SSA, RITMES, Marseille, France; 3 IHU-Méditerranée Infection, Marseille, France; 4 Institut de Recherche pour le Développement (IRD), Marseille, France; Natural History Museum, UNITED KINGDOM OF GREAT BRITAIN AND NORTHERN IRELAND

## Abstract

**Background:**

Urogenital schistosomiasis due to *Schistosoma haematobium* is a major public health problem in Mauritania, but little is known about its epidemiology in many areas of the country, particularly in the lake zones. The objectives of the present parasitological and malacological study were to assess the prevalence and intensity of urogenital schistosomiasis among school children in Kankossa and Oued Rawdha lakes, southern Mauritania, and determine the species of intermediate host snails and the prevalence of snails with schistosome.

**Methods:**

A school-based epidemiological survey was conducted in two villages in the lake areas of Kankossa and Oued Rawdha. Urine samples were collected from 450 state primary school children and Koranic school children and examined for the presence of *S*. *haematobium* eggs using filtration technique. Water bodies adjacent to human settlement were surveyed for *Bulinus* and *Biomphalaria* snails that may potentially be intermediate hosts of *S*. *haematobium*. Morphological, molecular, and proteomic (i.e. matrix-assisted laser desorption ionization time-of-flight mass spectrometry [MALDI-TOF MS]) identification of collected snails were conducted, and their infection status was assessed by real-time polymerase chain reaction (RT-PCR) using the highly repetitive *Dra*I gene.

**Results:**

The prevalence of urogenital schistosomiasis was 35.6% and 15.8% in Kankossa and Oued Rawdha villages, respectively, corresponding to ‘moderate’ prevalence (i.e., 10–49% infected schoolchildren). Urogenital schistosomiasis prevalence was higher in boys (30.0%) than in girls (21.2%; *P* < 0.05), and in Koranic schools pupils (37.1%) than in state schools (20.5%; *P* < 0.05) pupils. Multiple regression analysis showed that sex (odds ratio [OR]: 1.64; 95% confidence interval [95% CI]: 1.06–2.57; *P* = 0.03) and Koranic school level (OR: 1.79; 95% CI: 1.06–3.04; *P* = 0.03) were independently and significantly associated with urogenital schistosomiasis. Based on molecular and proteomic identification, both *B*. *senegalensis* and *B*. *umbilicatus* colonized the water bodies of Oued Rawdha, whereas both *B*. *forskalii* and *B*. *truncatus* colonized those of Kankossa. The *Dra*I RT-PCR detected *S*. *haematobium* complex DNA in 8 of 66 (12.1%) analysed snails: one *B*. *truncatus* and one *B*. *forskalii* in Kankossa and five *B*. *senegalensis* and one *B*. *umbilicatus* in Oued Rawdha.

**Conclusion:**

Urogenital schistosomiasis is moderately prevalent in the lake zones of Kankossa and, to a lesser extent, Oued Rawdha, located in southern Mauritania. Mass drug administration campaigns with praziquantel should be conducted to reduce the prevalence of urogenital schistosomiasis among school-aged children in the lake zone of Kankossa and Oued Rawdha village. Further parasitological and malacological studies should be conducted in other villages located in the Mauritanian lakes in the southern Sahelian zones and the northern oasis areas to strengthen our knowledge of the current epidemiological situation and implement appropriate urogenital schistosomiasis control strategies.

## Author summary

Urogenital schistosomiasis or bilharzia is caused by blood flukes transmitted in the urine. These parasites require compatible freshwater snails to develop and infect humans who come in contact with contaminated water. The adult females release thousands of eggs into the urinary tract, which may cause bloody urine, obstruction of urine flow, and many other urinary tract diseases. As in other developing countries, urogenital schistosomiasis is an important issue in Mauritania, where most previous studies on this disease have been concentrated along the Senegal River, the only permanent river in the country. In this work, we chose two places where schistosomiasis has never been studied before: Kankossa, where there is a permanent lake, and Oued Rawdha, where temporary ponds are present during part of the year. We found that 26% of school children had *Schistosoma* eggs in their urine, more in boys than girls, and more in younger children (6–8 years old) than in older children. We also identified the snail species responsible for disease transmission in these two sites. These data would be useful to organize mass treatment with appropriate medicines, kill the snails, disseminate the information to prevent community members from urinating in water bodies, and improve sanitary conditions.

## Introduction

Schistosomiasis is one of the most prevalent parasitic diseases after malaria and intestinal helminthiasis, with 251 million people worldwide requiring preventive chemotherapy in 2021 [1]. Approximately 90% of the world’s total cases of schistosomiasis occur in sub-Saharan Africa, where 200,000 deaths caused by, or associated with schistosomiasis are annually reported [2,3]. Schoolchildren aged 5–15 years old constitute a high risk group; they bear the highest burden of the disease which can cause both cognitive and physical impairments [4–6].

Urogenital and intestinal schistosomiasis, due to infection with *Schistosoma haematobium* and *Schistosoma mansoni* trematodes, respectively, are the most prevalent human schistosomiasis infections in sub-Saharan Africa [1,3]. Both *S*. *haematobium* and *S*. *mansoni* utilise freshwater snails as obligate intermediate hosts to reach the infective larval stage (cercariae) in freshwater bodies. Cercariae are shed from intermediate hosts and seek and penetrate the skin of definitive mammalian hosts. Humans who come in contact with contaminated freshwater become infected with *Schistosoma* spp. [7]. Freshwater snails belonging to the genera *Bulinus* and *Biomphalaria* are specific to the major human *Schistosoma* species found in Africa, *S*. *haematobium* and *S*. *mansoni*, respectively [8–12].

Schistosomiasis transmission is closely linked to agricultural development, which can expand the biotope of intermediate host snails and affect the distribution of predators that may control snail populations [13–17]. In Mauritania, the Senegal River is the only permanent river. In the Senegal River Basin (SRB), an increased number of snails has been reported following the construction of the Diama and Manantali dams in 1986 and 1989, respectively [18]. Since the parasitological and malacological surveys conducted in the early 1960s, animal (*Schistosoma bovis*) and human (*Schistosoma haematobium* and *Schistosoma mansoni*) *Schistosoma* spp., as well as *Bulinus* spp. and *Biomphalaria* spp., have been known to be present in different regions [19–21]. Several parasitological surveys conducted between the 1960s and 1980s confirmed the generally moderate to high prevalence of urogenital schistosomiasis in different regions of Mauritania [22]. In this context, schistosomiasis surveillance has been conducted periodically along both the Mauritanian and Senegalese sides of the banks of the Senegal River [23–32]. Malacological surveys conducted in or near the SRB showed the presence of several species of *Bulinus*, such as *B*. *truncatus*, *B*. *forskalii*, *B*. *senegalensis*, *B*. *globosus*, *B*. *umbilicatus*, *B*. *guernei*, and *B*. *jousseaumei*, in addition to *Biomphalaria pfeifferi* [4,21,23,24,33–36]. Parasitological surveys also showed a significant increase in the prevalence of schistosomiasis and the emergence of new foci [26,33,37,38].

Epidemiological data on schistosomiasis are scarce in areas apart from the SRB. The first nationwide survey for urogenital schistosomiasis was carried out in 1960 [20]. *Schistosoma haematobium*-positive urine samples were obtained from some local residents in the Saharan zone, but not from the Sahelian region of Hodh El Gharbi. In addition, *Bulinus* spp. (also *Biomphalaria* spp.) were collected from a large majority of these transmission foci, mostly in wells, waterholes, gueltas, and oases; some of the collected *Bulinus* snails (6–32%) emitted cercariae. In a sero-epidemiological survey conducted in 1973, the prevalence rates of seropositive children ranged from 1 to 76% in the age group 6–8 years old and 4 to 83% in the age group 9–18 years old [39]. Another parasitological survey dating back to the 1980s in Hodh El Gharbi region found *S*. *haematobium* infection in more than 50% of children under 16 years of age and peak prevalences ranging from 75% to 100% in children 6–10 or 11–15 years old [40].

Since 2001, praziquantel mass drug administration (PZQ MDA) has been the frontline control strategy of global schistosomiasis control strategies [41–44]. However, reinfections have been observed in children who had been successfully treated [45–48]. Reinfections after treatment are common due to the requirement of only a few *S*. *haematobium*-infected individuals to maintain the parasite’s life cycle, lack of potable water, low level of hygiene, sanitation, and health education resulting in the continued practice of contaminating water bodies and recreational activities involving contact with contaminated water that are difficult to control, as well as the long time interval between MDA [49–51]. These observations are indirectly supported by the results of a study conducted in Mauritania which showed that, two years after treatment, reinfection occurred in 35% of treated children [45]. In Mauritania, the first MDA campaign with praziquantel was implemented along the SRB in 2010 [30]. Nine additional MDA campaigns were implemented between 2012 and 2022, mostly along the SRB and in schoolchildren. However, recent prevalence surveys have shown that urogenital schistosomiasis remains endemic across all ecological zones of the SRB [26–30,32].

Malacological and parasitological investigations on schistosomiasis conducted in Mauritania have been mainly performed using traditional methods (i.e., macroscopic and microscopic morphological features). Microscopy suffers from several limitations, including low sensitivity and low specificity to correctly determine the parasite species based on parasite eggs and cercaria and to characterize the subspecies due to the presence of intraspecific morphological heterogeneity and potential natural hybridizations between different *Schistosoma* species [52,53]. Moreover, the accurate identification of *Bulinus* species using the snail’s shell morphology alone is hindered by the limited morphological differentiation within each species, thus requiring high-quality specimens and well-trained malacological experts [54,55]. At present, nucleotide-based identification of the parasite and snail species has become a useful approach to monitor the spread of schistosomiasis through sequencing nuclear or mitochondrial genes [56]. However, nucleotide-based approaches require technical training and capacity and are relatively expensive, which pose potential constraints in resource-limited settings. During the last decade, a novel proteomic tool, matrix-assisted laser ionization/desorption time-of-flight mass spectrometry (MALDI-TOF MS), has been developed and applied to identify microorganisms [57,58]. In advanced hospital laboratories where MALDI-TOF MS is available, this technology can replace traditional diagnostic methods for identifying microorganisms and also overcome many of the difficulties associated with the identification of fungi and bacteria [57,59]. In addition, this technology has wide application and has recently been used in malacology for the identification and classification of edible bivalve snails [60] and gastropods of medical importance, in particular the genera *Bulinus* and *Biomphalaria*, to species level [58,61]. MALDI-TOF MS is accurate and can be further improved by the addition of reference spectra validated by sequencing to the MS spectral database [57,62].

Reliable data on the intermediate hosts of *S*. *haematobium* are lacking in Mauritania. The present malacological and parasitological survey was conducted in two study sites in southern Mauritania to assess the prevalence and burden of urogenital schistosomiasis in children aged between 6 and 14 years old and identify the intermediate host snails using conventional, molecular, and proteomic tools. The usefulness of MALDI-TOF MS to differentiate between *Bulinus* species present in our study areas was assessed by comparing the results with those of PCR-sequencing as the reference method. The results of this survey will aid in the implementation of the most relevant control strategy in Assaba region [63].

## Materials and methods

### Ethics statement

Before enrolment, the objectives of the study were fully explained to the parents or legal guardians of the children in the dialect that they understand. After the explanation, the parents or legal guardians were given time for questions and signed an informed written consent form on behalf of their children, which was translated in local dialect. Only school children whose parents or legal guardians signed (or fingerprinted) the consent form participated in the study. The study protocol was reviewed and approved by the ethical review committee of the Mauritanian Ministry of Health, and the study was supervised by the coordinator of the Neglected Tropical Disease programme (059/MS/DGS). In the present study, children were not treated. The results of the survey were shared with the Mauritanian Ministry of Health for inclusion of the study sites for PZQ MDA.

### Study area

**Fig 1:**
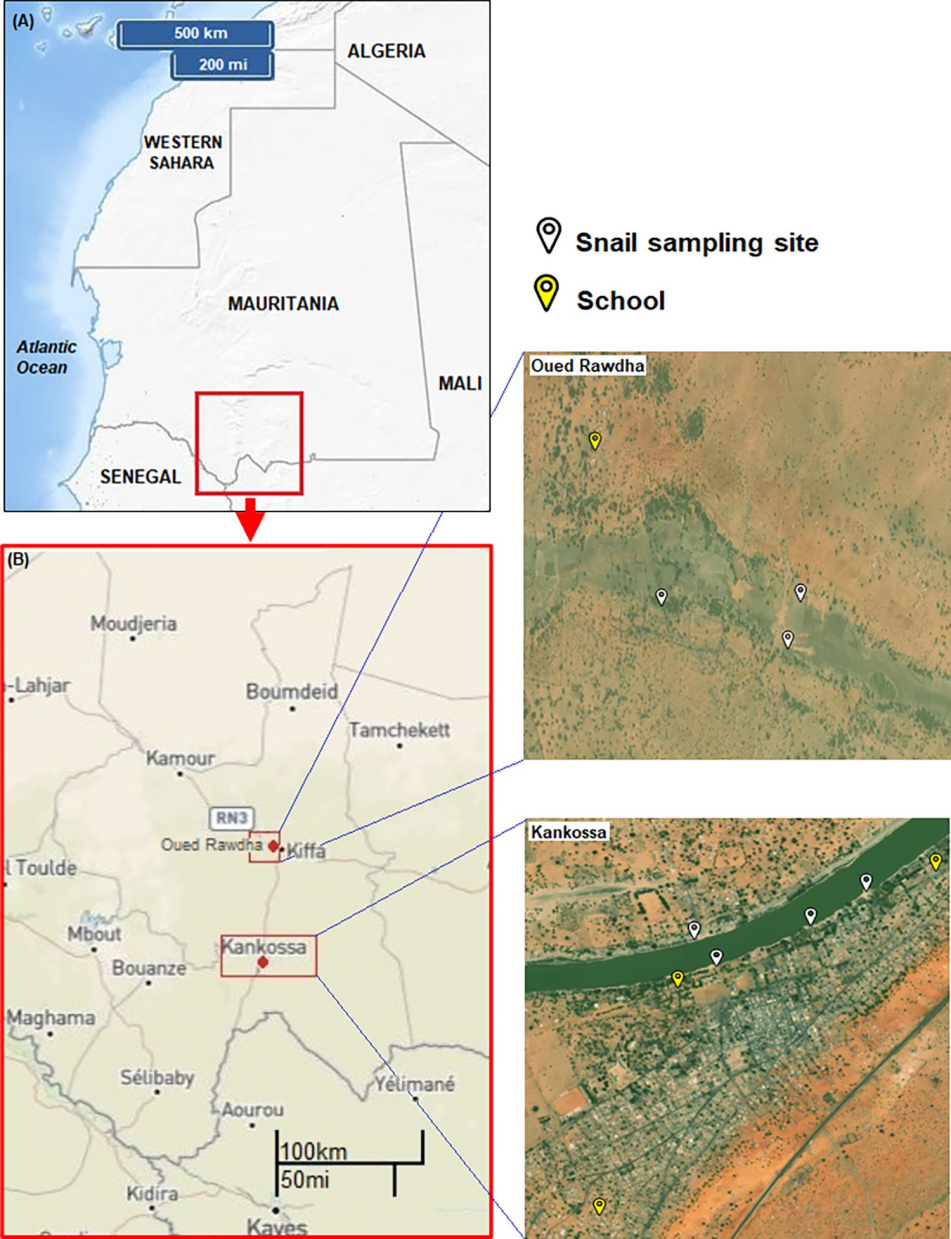
Map of the study sites in the lake zones of Kankossa and Oued Rawdha, Assaba region, southern Mauritania. The location of the collection sites of *Bulinus* snails (yellow arrow) and state schools (white arrows) is shown. Kiffa is the regional capital of Assaba. The Senegal River runs along the border between Senegal and Mauritania. The base layer for the map was sourced from: https://d-maps.com/carte.php?num_car=26766&lang=en. Fig 1 contains information from the public domain source: https://www.usgs.gov/.

The study was conducted in two villages of the Assaba region in southern Mauritania (Fig 1). Assaba is located between 16° and 17° north latitude and 17° and 25° west longitude, covering an area of 36,000 km^2^, corresponding to 3.5% of the surface area of Mauritania (i.e., a total of 1,085,000 km^2^). The total population of the region was 405,389 inhabitants in 2022 [64], and 48% were children under 15 years old. Assaba is one of the poorest regions in Mauritania with an overall poverty incidence of 67%, reaching 83% in rural areas of the region. Kiffa (16°37’N; 11°24’W), the regional capital of the Assaba, is the third largest city in the country with 110,715 inhabitants, according to the latest available population census in 2013 [65].

The majority of children aged between 6 and 12 years attend state primary schools. In addition to formal state schools, non-formal Koranic schools, a neighbourhood institution concentrated on religious education, are widespread across Mauritania. There are no data on the number of Koranic schools in the Assaba region, but they are attended by children who have either abandoned their education or have not been to a formal school, with a maximum age up to 14 years old, and also by those who attend a formal school, either after school or during vacations. The grade levels in primary schools range from 1 to 6, roughly corresponding to first to sixth grades in the American educational system. Most school children in the first grade (referred to as ‘level 1’ in this paper) are approximately 6 years old; second grade (level 2), 7 years old; third grade (level 3), 8 years old; fourth grade (level 4), 9 years old; fifth grade (level 5), 10 years old; and sixth grade (level 6), 11 years old. There is no rigid structure with distinct age groups in Koranic schools in the country. For statistical purposes, children who are exclusively in Koranic schools were considered to be at level 0.

Kankossa (15°55’N; 11°31’W) is one of the five departments of the Assaba region (Fig 1). Kankossa city is located approximately 80 km south of the regional capital, Kiffa, along a permanent lake called Kankossa Lake. The lake, ~7.5 km^2^, depending on the season, is one of the largest permanent water bodies in Mauritania. The lake receives water from rainfall, as well as from other smaller lakes in the surrounding area (Lahraj lake, Ajar lake, Loubkherée) through oueds or wadi (defined as stream bed that remains dry through most of the year, except during the rainy season) [66,67]. It is classified as an “open lake;” its water leaves the lake by a dry river called Karakoro oued or wadi and joins the Senegal River. Kankossa department has 82494 inhabitants (approximately 11,000 inhabitants in Kankossa city, the capital of the department of Kankossa) and houses six state primary schools (called “Ecole 1” to “Ecole 6” or “School 1” up to “School 6” in English) and several Koranic schools [65]. The three schools selected in the present study, “Ecole 1”, “Ecole 2”, and “Ecole 5”, host over 1,100 pupils. The main occupations of the inhabitants are subsistence farming (market gardening, cultivation of principal staples, i.e., millet and sorghum), or livestock farming by nomadic pastoralists. Climate is Sahelian, with 300 to 400 mm annual rainfall. Mean annual temperature and relative humidity are 33°C and 55%, respectively [68].

The small village of Oued Rawdha (16°40’N; 11°26’W) is located approximately 8 km North West of Kiffa on the edge of a 15 km-long basin of the same name (Fig 1). Oued Rawdha basin is a seasonal stream that supplies water for market gardening and oasis crops during the cold season. The total population of the village is approximately 2,000 residents. It has only one state primary school, called Zoubeir Ibn Alawam, hosting 242 pupils and few Koranic schools. The mean annual rainfall is 300 mm. Average minimum and maximum temperatures are 23.6°C and 37.1°C, respectively, while average minimum and maximum humidity are 24% and 54%, respectively [68]. Rain fed agriculture and livestock farming are the main economic activities of Oued Rawdha villagers. PZQ MDA campaigns have never been implemented before in our two study sites.

### Study design and sample size

Cross-sectional parasitological and malacological surveys were conducted in 2022 in the villages of Kankossa and Oued Rawdha during both the dry (October to June) and rainy (July to September) seasons [69]. A total of 450 schoolchildren were enrolled (n = 232 from Kankossa and n = 218 from Oued Rawdha). The inclusion criteria were age between six and 14 years, residence in the village for at least six months, no less than six months PZQ treatment history (based on questionnaire), and written consent of the parents to participate in the study. Randomization was not done. The basic socio-demographic information was obtained from each participant and recorded in an anonymized questionnaire. The questionnaire consisted of questions about their identity (first name and family name; later anonymized), age, school, class to determine their educational level, recreational activities in water bodies, including the number of times they went to a water body per day and the exact location where they go to in the lake or stream, whether they pass or had passed red colored urine before, basic knowledge about snails (photographs of snail specimens were shown) and urogenital schistosomiasis, and recent treatment with PZQ.

The sample size of the study was calculated using the following formula:

$$N=\frac{\varepsilon^{2}p(1-p)}{d^{2}}$$

where *N* is the minimum sample size, ε is the normal *Z* score corresponding to the standard normal distribution (ε = 1.96, 95% confidence interval [CI]), *p* is the estimated prevalence (it was set at 50% because no previous studies had been carried out in the area and similar studies along the SRB found the prevalence rates of 30.1–57.4% [26,29,30]), and *d* is the margin of error set at 5%. The number of children to be included was 450 children, considering a 95% confidence level, a 5% margin of error and an expected non-response rate (defined in this study as schoolchildren whose parents provided their consent to participate in the study but were absent on the day of survey or refused to provide their urine sample) of 15%.

### Urine collection and examination

Urine samples were collected in a 120-ml sterile container from each child during the day between 10:30 and 14:00 when schistosome egg excretion is known to be highest [70]. Each container was labelled with unique identifier before immediately examining the appearance of the urine through a transparent plastic container.

The urine samples in the containers were placed in an icebox (without ice) to prevent a spill and immediately transported to the nearby clinical laboratory of either Kankossa health center (for samples collected in Kankossa) or the Hospital Center of Kiffa (for samples collected in Oued Rawdha). Due to the proximity between the study sites and their respective clinical laboratories, it took less than 15 min to transport the samples from different schools to the laboratories. Upon arrival to one of the collaborating laboratories, urine samples were immediately resuspended and filtered according to the standard WHO procedure to detect presence of *S*. *haematobium* eggs and assess the intensity of infection [71]. Briefly, 10 ml of urine from each child were filtered through a Nytrel microfilter membrane (pore size between 10–12 μm; Vestergaard Frandsen Group, Kolding, Denmark) using a sterile syringe. The filter membrane was examined under a microscope for schistosome eggs under 40× magnification. The quantity of *Schistosoma* eggs was expressed as the number of eggs/10 ml of urine. The intensity of *S*. *haematobium* infection was expressed as the number of eggs/10 ml of urine and classified as ‘light’ (1–49 eggs/10 ml urine) or ‘heavy’ (≥ 50 eggs/10 ml urine) according to the WHO recommendations [63].

### Malacological study

#### Freshwater snail collections

In Kankossa, snails were collected from the permanent lake and streams that feed the lake while they were collected from temporary ponds in Oued Rawdha. The collection points in both villages were identified based on the information collected during the interviews held with children and corresponded to the areas where villagers carry out their domestic chores or fetch water and children play in the water. Based on this information, snail collection was performed for three consecutive days in the morning (8:00 to 10:00) at four sites in Kankossa (two investigators collected the snails) and three sites in Oued Rawdha (one investigator collected the snails) during the dry and rainy seasons. The snails were collected from the vegetation surrounding the water points, including branches and dead leaves, by hand when possible or by using a standard scoop net. Rain boots and hand gloves were used during sampling to protect the investigators from possible infection by cercariae. Snails collected from the same water point were grouped together in the same pre-labelled container.

Snail morphological identification was based on shell morphology using standard taxonomic keys [72]. The specimens were examined under a magnifying glass. The collected snails from each study site were initially separated according to their genus; those that did not belong to *Bulinus* spp. were not further analyzed. The snails were then classified by species and geographical origin and preserved in 70% ethanol for molecular and proteomic analyses.

#### DNA extraction and nucleotide sequence analysis

Two to four specimens of each morphologically identified *Bulinus* species were randomly selected for DNA-based identification. Genomic DNA was extracted from the tissue of individual snails (with the exception of the foot which was used for MALDI-TOF MS analysis) using EZ1 DNA Tissue Kit (Qiagen, Hilden, Germany) according to the manufacturer’s instructions. Snail tissue was incubated at 56°C overnight with 180 μl of G2 lysis buffer and 20 μl of proteinase K until cells were lysed, and tissue samples were homogenized. DNA extraction from pre-treated tissue was performed using the EZ1 BioRobot Extraction Device (Qiagen Hilden, Germany). Total DNA from each sample was eluted with 200 μl of ethylenediamine tetraacetic acid (EDTA) buffer and stored at -20°C until use.

Two molecular targets were chosen for polymerase chain reaction (PCR) amplification and sequencing: the mitochondrial cytochrome oxidase subunit I gene (COI) and ribosomal 28S rRNA. The PCR amplification targeting a 710-base pair (bp) fragment of COI and 588 bp region of the 28S ribosomal subunit was carried out using a thermocycler (Applied Biosystems, Foster City, CA, USA). PCR was performed in a reaction mixture consisting of 2 μl of extracted snail DNA, 12.5 μl of AmpliTaq Gold 360 PCR master mix (Applied Biosystems, Waltham, MA, USA), 0.5 μl of each primer (at 20 μM), and 9.5 μl of sterile distilled water, in a final reaction volume of 25 μl.

The COI region was amplified using Folmer’s universal primers LCO1490, HCO2198 [73], and forward Bglob-CoxF primer [74]. The forward primer BglobCoxF was used to sequence *Bulinus globosus*. The thermal cycler was programmed as follows: an initial denaturation at 95°C for 15 min, followed by 40 cycles of denaturation at 95°C for 30 s, hybridization at 40°C for 30 s, and extension at 72°C for one minute 30 s, and a final extension at 72°C for 7 min.

The fragment of 28S ribosomal unit was amplified with forward 28SF4 and reverse 28SR5 primers [75]. The thermal cycler was programmed as follows: an initial denaturation at 95°C for 15 min, followed by 39 cycles of denaturation at 95°C for 30 s, hybridization at 55°C for 50 s, and extension at 72°C for one minute 30 s, and a final extension at 72°C for 7 min. Primer sequences used to perform PCR are presented in S1 Table.

The amplified products were purified using a Macherey Nagel plate (NucleoFast 96 PCR, Düren, Germany). For each amplified fragment, Sanger sequencing was performed in both 5’-3’ and 3’-5’ directions using the same primers as for PCR with BigDye Terminator v1.1, v3.15× sequencing buffer (Applied Biosystems, Warrington, UK) and run on an ABI 3100 automated sequencer (Applied Biosystems). The sequences were assembled using Chromas Pro software, version 1.7.7 (Technelyium Pty. Ltd, Tewantin, Australia), and different alignments were performed with BioEdit software (http://www.mbio.ncsu.edu/BioEdit/bioedit.html) using the ClustalW algorithm to perform multiple alignments of our sequences, accounting for insertions and deletions while optimizing the correspondence between the sequences. The correction of sequences consisted of manually editing the sequences to detect and correct sequence errors and ambiguous nucleotides based on the visual inspection and analysis of the chromatograms using Chromas Pro version 1.7.7 software. Low-quality sequence segments located at the ends of the sequencing reads were trimmed to avoid errors in subsequent analyses. After sequence correction, analysis with basic local alignment search tool (BLAST) was performed on the National Centre for Biotechnology Information (NCBI) online database for species identification based on the sequence data from all three genes (i.e., 28S ribosomal unit, COI, and Bglob-cox).

Phylogenetic tree was constructed using the maximum likelihood (ML) statistical method. For this analysis, the substitution model chosen was the Tamura-Nei model with a gamma distribution (TN93+G) (i.e., an extension of Kimura’s two-parameter distance model), which provides a more realistic model of the processes of molecular evolution and gives a better estimate of the phylogenetic relationships based on our DNA sequences. The selection of the most appropriate substitution model for constructing the tree was determined by Molecular Evolutionary Genetics Analysis (MEGA) v7.0.26 software using the “Find Best DNA/Protein Models” option [76,77]. This software first evaluates several substitution models and selects the one that best matches our sequence data to ensure that the phylogenetic tree accurately reflects the evolutionary relationships based on our analysed nucleotide sequences. The following reference sequences were obtained from GenBank and used in phylogenetic analysis: *B*. *senegalensis*, OP811205 and OP811206; *B*. *umbilicatus*, OP811201 and OP811202; *B*. *truncatus*, AF435659; *B*. *forskalii*, AF435655; *B*. *globosus*, OP811203 and OP811204; *B*. *ugandae*, AF435660; *B*. *africanus*, AF435658; *B*. *nasutus*, AF435656; and *Lanistes varicus*, OR339575. The statistical support of the inner branches of the trees was assessed by bootstrapping with 1000 iterations.

#### Molecular screening of snails for *Schistosoma* spp. infection

The detection of *S*. *haematobium*-group infection in the extracts of snail genomic DNA was performed using the real-time PCR (RT-PCR) technique. The reaction targets the sequence of the highly repetitive *Dra*I sequence (120 bp) of the *S*. *haematobium* group using the primer pairs Sh1 and Sh2 previously described by Hamburger et al. [78] and the fluorescent labelled hydrolysis probe developed by Cnops *et al*. [79]. The primer sequences and probe used to perform RT-PCR are presented in S1 Table.

The RT-PCR amplification was performed in a 20-μL reaction mixture containing 5 μL of DNA, 10 μL of the Roche Master Mix (Roche; Basel, Switzerland), 3.5 μL of sterile distilled water, and 0.5 μL of each primers (20 μM), and 0.5 μL of the probe (50 μM; Applied Biosystems, Foster City, CA, USA). RT-PCR was performed using a CFX96 thermal cycler (Bio-Rad, Marnes-la-Coquette, France). The thermal programme was as follows: an initial 2-min denaturation step at 95°C, followed by a 3-minute denaturation step at 95°C, then 40 cycles of 95°C for 30 s and 60°C for 1 min, followed by 4°C indefinitely. The RT-PCR result was considered positive when the cycle threshold (Ct) value was less than 35 [79]. For each PCR run, a negative control (distilled water) and positive control (DNA from *S*. *haematobium* eggs identified by sequencing in an earlier study performed in Senegal [61]) were used.

#### MALDI-TOF MS analysis

The snail foot was selected for the optimization of MALDI-TOF MS analysis as it constitutes the best protein source compared to other body parts [80]. The feet were dissected with a sterile surgical knife, rinsed thrice in distilled water, cut into small pieces, and dried before protein extraction according to the protocol described by Hamlili *et al*. [58]. Briefly, each piece of dissected foot was individually placed in a 1.5 ml microfuge tube with glass beads ≤ 106 μm (Sigma Aldrich, Lyon, France) and 30 μl of an extraction mix solution containing 70% (v/v) formic acid, 50% (v/v) acetonitrile, and water for high-performance liquid chromatography (HPLC). Samples were homogenized using a TissueLyser II device (Qiagen, Hilden, Germany) with optimized parameters (three 1-minute cycles at a frequency of 30 Hertz) [81]. After a brief centrifugation at 2000×*g* for 30 s, protein extracts were spotted in four replicates on the MALDI-TOF MS target plate (Bruker Daltonics, Wissembourg, France). The extracts were covered with 1 μl of matrix suspension composed of saturated α-cyano-4-hydroxy cinnamic acid (CHCA) (Sigma, Lyon. France), 50% acetonitrile (v/v), 2.5% trifluoroacetic acid (v/v) (Aldrich, Dorset, UK), and HPLC-grade water. The mixture was left to dry at room temperature for 10 min before analysing the samples with MALDI-TOF Microflex mass spectrometer (Bruker Daltonics, Bremen, Germany). Protein mass spectra were obtained using a Microflex MALDI-TOF Mass Spectrometer (Bruker Daltonics) with flex Control v.2.4 software (Bruker Daltonics). The spectral profiles obtained from snail feet were visualized using flexAnalysis software version 3.3 (Bruker Daltonics). For spectral data processing (smoothing, base subtraction, and peak selection), plot analysis, dendrogram and principal component analysis (PCA), spectra were exported into ClinProTools v.2.2 and MALDI-Biotyper v.3.0 (Bruker Daltonics, Germany).

#### Creation of a reference spectra database

To improve our database of reference spectra for freshwater snails, a subset of snail specimens identified morphologically and confirmed by DNA-based sequencing were characterized by MALDI-TOF MS. The quality of MALDI-TOF spectra was confirmed using flexAnalysis v.3.3 software. Spectra of poor quality (peak intensity < 3 000 arbitrary units (a.u.) and/or non-reproducible) were excluded. The intraspecific reproducibility and interspecific specificity were evaluated by comparing the spectral profiles of each specimen using gel visualization, dendrogram, and PCA functions of ClinProTools v2.2 and MALDI-Biotyper v3.0 software (Bruker Daltonics).

To update the database of reference spectra, a dendrogram was created using the MALDI-Biotyper v3.0 dendrogram function to visualize the similarity or distances between selected MS spectra selected using flex Analysis v.3.3 software (Bruker Daltonics).

#### Blind tests

The spectra obtained from new specimens (n = 54) (other than those used to create the MS database) were compared from the updated reference spectra database using MALDI-Biotyper v3.0 software. The degree of similarity between the query and reference spectra from the database was estimated via log score values (LSV) ranging from 0 to 3, depending on the level of matching. The identification was correct when the LSV was > 1.7 [58].

#### Data analysis

The prevalence of schistosomiasis was calculated overall and stratified by study site, age, sex, and type of school. The data obtained from the survey were entered into Microsoft Excel 2010 spreadsheet (Microsoft Corp., Redmond, WA, USA) and analysed with RStudio version 2022.07.2 and R version 4.3.0 softwares [82,83]. Infection prevalence rates were compared by using the chi-square test. Multivariate logistic regression was used to assess the associations between urogenital schistosomiasis and socio-demographic factors (age, sex, and education level). Odds ratios (ORs) were calculated to assess the risk factors (age, sex, and school type, i.e. state school vs Koranic school) associated with urogenital schistosomiasis. Associations were considered statistically significant when *P* values were less than 0.05. Factors with *P* value <0.05 in the univariate analysis were included in the multivariate analysis. The R packages and functions used to analyze our data are described in (S1 Appendix).

## Results

### Sociodemographic characteristics of the children

A total of 450 school-age children were screened for urogenital schistosomiasis in two villages (Table 1). Of the included children, 232 (51.5%) were from Kankossa village and 218 (48.5%) were from Oued Rawdha village. None of the included children had taken praziquantel. This result was expected because mass drug administration programme had never been implemented in Kankossa and Oued Rawdha in the past. The sex ratio (M/F) was 1.07, with 233 males (51.8%) and 217 females (48.2%). The age ranged from 6 to 14 years, with a mean age (± standard deviation [SD]) of 9.5 (± 2.5) years (S1 Data). The number of children aged 6–8 years was significantly higher than the other age groups (chi-square = 22.173, df = 2, *P* = 0.0001). Of 450 enrolled children, 307 (68.2%) were recruited in state primary schools, and 143 (31.8%) were from Koranic schools. Among the children in state primary schools, 151 (33.6%) were in levels 1 to 3, and 156 (34.7%) were in levels 4 to 6.

**Table 1:** Characteristics of the study participants in the lake zones of Kankossa and Oued Rawdha, Assaba region, southern Mauritania.

Characteristics		N	Proportion (%)	*P*-value
Study site				
	Kankossa	232	51.5	0.51
	Oued Rawdha	218	48.4	
Sex				
	Male	233	51.8	
	Female	217	48.2	
	Sex ratio	1.07	--	
Age group (years)				
	6–8	197	43.8	0.0001
	9–11	129	28.7	
	12–14	124	27.5	
	Mean (± SD)	9.5 (2.5)	--	
School type				
	Koranic	143	31.8	< 0.0001
	Primary	307	68.2	
School level*				
	0 (Koranic school)	143	31.8	0.81
	1–3	151	33.6	
	4–6	156	34.7	

N = number of children (total = 450). SD, standard deviation.

* Informal Koranic school level, noted 0, includes children from all age group (6–14 years old). Children in the primary school (state school) levels 1, 2, and 3 (corresponding to first, second, and third grades in the American educational system) are those aged approximately 6, 7, and 8 years old, respectively, while children in levels 4, 5, and 6, corresponding to fourth, fifth, and sixth grades in the American educational system, are those aged approximately 9, 10, and 11 years old, respectively.

#### *S*. *haematobium* infection prevalence and intensity

In this study, all 450 urine samples provided by school-aged children were examined for the presence of *S*. *haematobium* eggs. Overall, the prevalence of urogenital schistosomiasis was 25.8% among the enrolled children. The prevalence was significantly higher in school children from Kankossa (35.3%; 82/232) than those from Oued Rawdha village (15.6%; 34/218; *P* < 0.0001) (Table 2). Prevalence in boys (30%; 70/233) was significantly higher than in girls (21.2%; 46/217) (chi-square = 4.54, df = 1, *P* = 0.0331). There was a statistically significant negative correlation between age and prevalence of infection (*P* = 0.004). The school children in age group 6–8 years were the most infected (33%; 65/197) compared to those of other age groups (9–11 years old and 12–14 years old). Children from Koranic schools had a higher prevalence of infection (37.1%), compared to those from state primary schools (20.5%; chi-square = 14.02, df = 1, *P* = 0.0002). The prevalence of *S*. *haematobium* infection was 19.1% (65/340) and 46.3% (51/110) in the urine samples collected and examined during the dry and wet seasons, respectively (Table 2). Of the 116 positive urine samples, 68 (58.6%) had a light (1–49 eggs/10 ml) infection whereas 48 (41.4%) had a heavy (≥ 50 eggs/10 ml) *S*. *haematobium* infection (*P* = 0.069).

**Table 2:** Prevalence and risk factors of *S*. *haematobium* infection in the lake areas of Kankossa and Oued Rawdha, Assaba region, southern Mauritania.

Variable		No. tested	*Schistosoma haematobium* infection		*P-value*
			Positive	Prevalence (95% CI)	
Total		450	116	25.8 (22.0–30.0)	
Study village					
	Kankossa	232	82	35.3 (29.5–41.7)	<0.0001
	Oued Rawdha	218	34	15.6 (11.4–21.0)	
Sex					
	Male	233	70	30.0 (24.5–36.2)		0.0321
	Female	217	46	21.2 (16.3–27.1)	
Age group (years)					
	6–8	197	65	33.0 (26.8–39.8)	0.0004
	9–11	129	29	22.5 (16.1–30.4)	
	12–14	124	22	17.7 (12.0–25.4)	
School type					
	Koranic school	143	53	37.1 (29.6–45.2)	0.0002
	Primary school	307	63	20.5 (16.4–25.4)	
School level*					
	Koranic School (level 0)	143	53	37.1 (29.6–45.2)	0.0004
	Primary School (levels 1–3)	151	36	23.8 (17.7–31.2)	
	Primary School (levels 4–6)	156	27	17.3 (12.2–24.0)	
Season					
	Dry	340	65	19.1 (15.1–23.7)	<0.0001
	Wet	110	51	46.3 (36.8–56.1)	
Infection intensity**					
	Light (1–49 eggs/10 ml urine)	-	68	58.6 (49.1–67.7)	0.0691
	Heavy (≥ 50 eggs/10 ml urine)	-	48	41.4 (32.3–50.9)	

* Informal Koranic school level, noted 0, includes children from all age group (6–14 years old). Children in the primary school (state school) levels 1, 2, and 3 (first, second, and third grades in the American educational system) are those aged approximately 6, 7, and 8 years old, respectively, while children in levels 4, 5, and 6, corresponding to fourth, fifth, and sixth grades in the American educational system, are those aged approximately 9, 10, and 11 years old, respectively.

**Proportions of positive results denote the number of light/heavy infections samples among total positive samples (n = 116).

### Intensity of infection by study village

**Fig 2:**
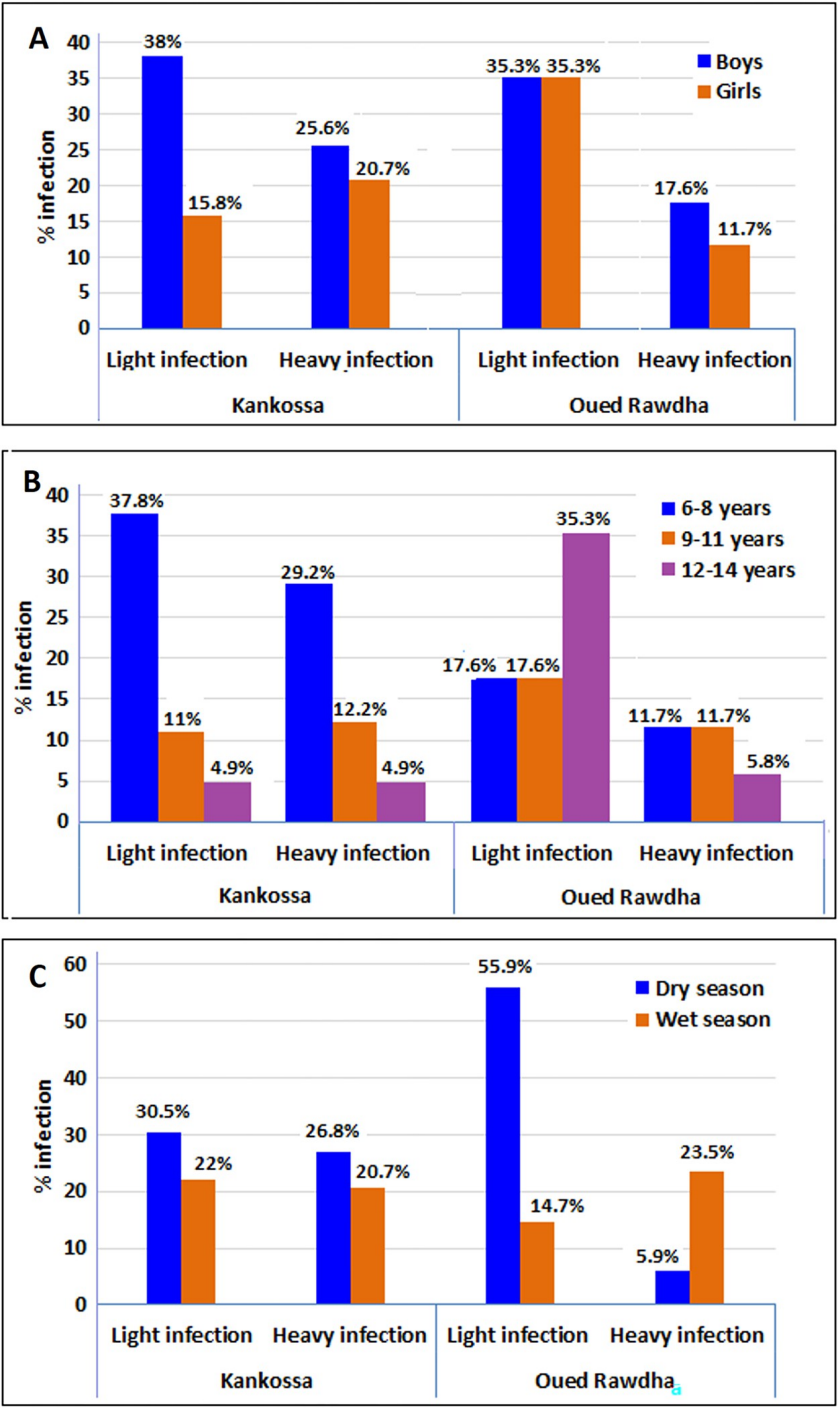
Prevalence and intensity of *S*. *haematobium* infection according to sex [A], age groups [B], and season [C] among school-age children in the lake zones of Kankossa and Oued Rawdha, Assaba region, southern Mauritania. The intensity of infection was classified as light (1–49 eggs/10 ml urine) and heavy (≥ 50 eggs/10 ml urine), according to the WHO classification [63].

The intensity of *S*. *haematobium* infection expressed as the number of *Schistosoma* eggs in 10 ml urine was assessed by sex, age, and season in the two study villages (Fig 2 and S2 Data). Of the 116 positive urine samples, 68 (58.6%) had a light (1–49 eggs/10 ml) infection whereas 48 (41.4%) had a heavy (≥ 50 eggs/10 ml) *S*. *haematobium* infection (*P* = 0.069). A higher proportion of children had a light infection during the dry season (64.7%; 44/68) compared to the wet season (35.3%; 24/68; chi-square = 5.33, df = 1, *P* = 0.021). No statistically significant differences in the intensity of infection among boys and girls in each village (chi-square = 0.15, df = 1, *P* = 0.15) and between the two villages (chi-square = 1.21, df = 1, *P* = 0.27) were observed. Similar trends were observed within and between the two study villages when the intensity of the urogenital schistosomiasis (light vs. heavy infections) was considered by age groups and season (Fig 2). The proportion of heavy intensity of the infection was slightly higher during the dry season, compared to the rainy season, in Kankossa (26.8% versus 20.7%, respectively), but an opposite trend was observed in Oued Rawdha (5.9% during the dry season versus 23.5% during the rainy season).

### Socio-demographic risk factors of *S*. *haematobium* infection

The results of the multivariate analysis of the factors associated with urogenital schistosomiasis are presented in Table 3. The age of children was not a risk factor (*P* > 0.05), whereas the odds of infection increased significantly in boys compared with girls and in children attending Koranic schools compared with state primary schools, respectively (*P* < 0.05).

**Table 3:** Multivariate analysis of risk factors associated with urogenital schistosomiasis among school children in the lake areas of Kankossa and Oued Rawdha, Assaba region, southern Mauritania.

Factor	Variable	OR	95% CI	*P*-value
Sex	Female	1	–	–
	Male	1.64	1.06–2.57	0.03 *
Age (years)	6–8	1	–	–
	9–11	0.75	0.42–1.35	0.34
	12–14	0.58	0.31–1.07	0.08
School type	Primary school	1	–	–
	Koranic school	1.79	1.06–3.04	0.03 *

OR, odds ratio; 95% CI, 95% confidence interval

*statistically significant association (*P* < 0.05).

### Snail collection and morphological identification

A total of 231 freshwater snail specimens (59 from Kankossa and 172 from Oued Rawdha) were collected from different water points. Based on the key morphological features, they were identified as *Bulinus truncatus* (n = 32), *B*. *umbilicatus* (n = 40), *B*. *globosus* (n = 33), *B*. *forskalii* (n = 56), and *B*. *senegalensis* (n = 70) (Table 4). All live *Bulinus* snails from both study sites were collected during the rainy season (July to September). *Bulinus* snails were either not found (in Oued Rawdha) or found dead (i.e. empty shells, in Kankossa) during the dry season (October to June). Other *Bulinus* spp. previously found in or near the SRB, namely *B*. *guernei* and *B*. *jousseaumei* [23,24], were not found in Kankossa and Oued Rawdha. Likewise, *Biomphalaria* snails reported earlier in the SRB were not present in our study areas [33].

**Table 4:** Morphological identification of *Bulinus* snails collected during the rainy season in 2022 in Kankossa and Oued Rawdha.

Snail species	Number of *Bulinus* snails (n, %)	
	**Kankossa (n = 59)**	**Oued Rawdha (n = 172)**
*Bulinus truncatus*	17 (28.8)	15 (8.7)
*Bulinus umbilicatus*	16 (27.1)	24 (14.0)
*Bulinus globosus*	10 (16.9)	23 (13.4)
*Bulinus forskalii*	1 (1.7)	55 (32.0)
*Bulinus senegalensis*	15 (25.4)	55 (32.0)

Live *Bulinus* snails were found only during the rainy season (July to September). During the dry season (October to June) in 2022, *Bulinus* snails collected in Kankossa were all dead (only empty snail shells were collected); in Oued Rawdha, *Bulinus* snails were not found.

### DNA-based identification of snails

**Fig 3:**
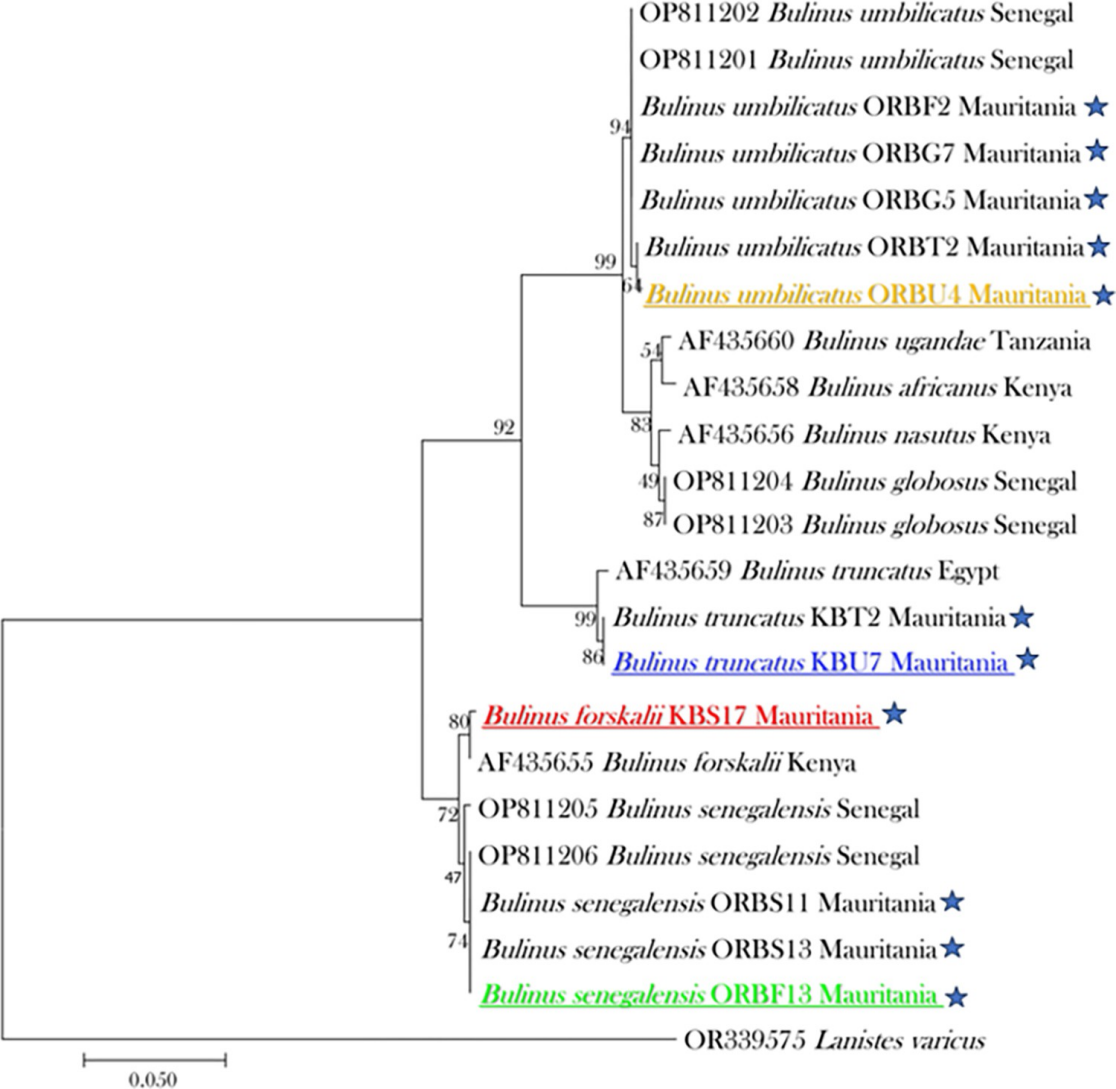
Phylogenetic tree based on the nucleotide sequence of a 588-base pair region of the 28S rRNA gene, constructed using maximum likelihood method based on the TN93+G substitution model with MEGA 7. The values on the branches are bootstrap support values based on 1000 replicates. The asterisks indicate the specimens collected and analysed in the present study with their corresponding GenBank accession numbers. The other *Bulinus* snails are reference specimens with their GenBank accession number and geographic origin.

DNA-based identification of the snails was performed using the COI (710 bp) of the mitochondrial genome and the 28S rRNA region (588 bp) of the nuclear genome, on 12 randomly selected specimens from each of five species initially determined by morphological identification keys, notably, *B*. *senegalensis* (n = 3), *B*. *forskalii* (n = 3), *B*. *umbilicatus* (n = 2), *B*. *truncatus* (n = 2), and *B*. *globosus* (n = 2). The DNA-based identification of these 12 specimens enabled us to confirm their morphological description in order to update our MALDI-TOF MS database after obtaining their spectral fingerprint. The BLAST analysis of both the COI and 28S rRNA sequences yielded high identity scores ranging from 98.0% to 100%, as summarized in Table 5. Some discrepancies between morphological and molecular identification were observed, notably specimens morphologically identified as *B*. *senegalensis* (n = 3) were identified as *B*. *forskalii* based on 28S rRNA sequence that shared 100% identity with GenBank accession number AF435655. Similarly, for *B*. *forskalii*, *B*. *umbilicatus*, and *B*. *truncatus* (as initially identified based on morphological keys), the correct molecular identification of one specimen of each of these snail species was determined using 28S rRNA as *B*. *umbilicatus* (GenBank accession number OP811202), *B*. *truncatus* (GenBank accession number AF435659), and *B*. *umbilicatus* (GenBank accession number OP811202), respectively. The identification of two specimens of *B*. *globosus*, initially identified based on morphological criteria, was corrected to *B*. *umbilicatus* (GenBank accession number OP811202) after 28S rRNA sequencing. The same observation was made with the COI region, and there was no discordance between COI- and 28S rRNA-based speciation, as detailed in Table 5. All sequences obtained in the present study were deposited in the GenBank database with accession numbers OR921249–OR921253 (for the fragment of 28S ribosomal unit) and OR921236–OR921239 (for the COI region) (S2 Appendix). The phylogenetic tree based on the 28S rRNA sequences showed a clear interspecific discrimination (Fig 3). The GenBank accession numbers of the reference sequences are presented in Fig 3.

**Table 5:** *Bulinus* snail identification based on mitochondrial and nuclear DNA sequences.

Morphological ID	Mitochondrial genome (N = 12)		Nuclear genome (N = 12)		DNA-sequence based ID
	COI (% identity) (n = sequences)	GB accession no.***	28S rRNA (% identity) (n = sequences)	GB accession no.***	
*B*. *senegalensis* (n = 3)	*B*. *senegalensis* (99.4%)	OP811028.1	*B*. *senegalensis* (99.8%)**	OP811206.1	*B*. *senegalensis (n = 2)* *B*. *forskalii (n = 1)*
	NA		*B*. *forskalii* (100%)**	AF435655.1	
*B*. *forskalii* (n = 3)	*B*. *senegalensis* (98.4%)	MW167056.1	*B*. *senegalensis* (98.8%)**	OP811206.1	*B*. *senegalensis (n = 2)* *B*. *umbilicatus (n = 1)*
	NA		*B*. *umbilicatus* (100%)**	OP811202.1	
*B*. *umbilicatus* (n = 2)	*B*. *umbilicatus* (98.7%)	OP811022.1	*B*. *umbilicatus* (99.2%)**	OP811202.1	*B*. *umbilicatus (n = 1)* *B*. *truncatus (n = 1)*
	*B*. *truncatus* (99.8%)**	MG407296.1	*B*. *truncatus* (99.7%)	AF435659.1	
*B*. *truncatus* (n = 2)	*B*. *truncatus* (100%)**	MG407285.1	*B*. *truncatus* (99.6%)	AF435659.1	*B*. *truncatus (n = 1)* *B*. *umbilicatus (n = 1)*
	*B*. *umbilicatus* (98.0%)	OP811022.1	*B*. *umbilicatus* (99.2%)**	OP811202.1	
*B*. *globosus* (n = 2)	*B*. *umbilicatus* (98.6%)	OP811022.1	*B*. *umbilicatus* (99.2%)**	OP811202.1	*B*. *umbilicatus (n = 2)*

**Fig 4:**
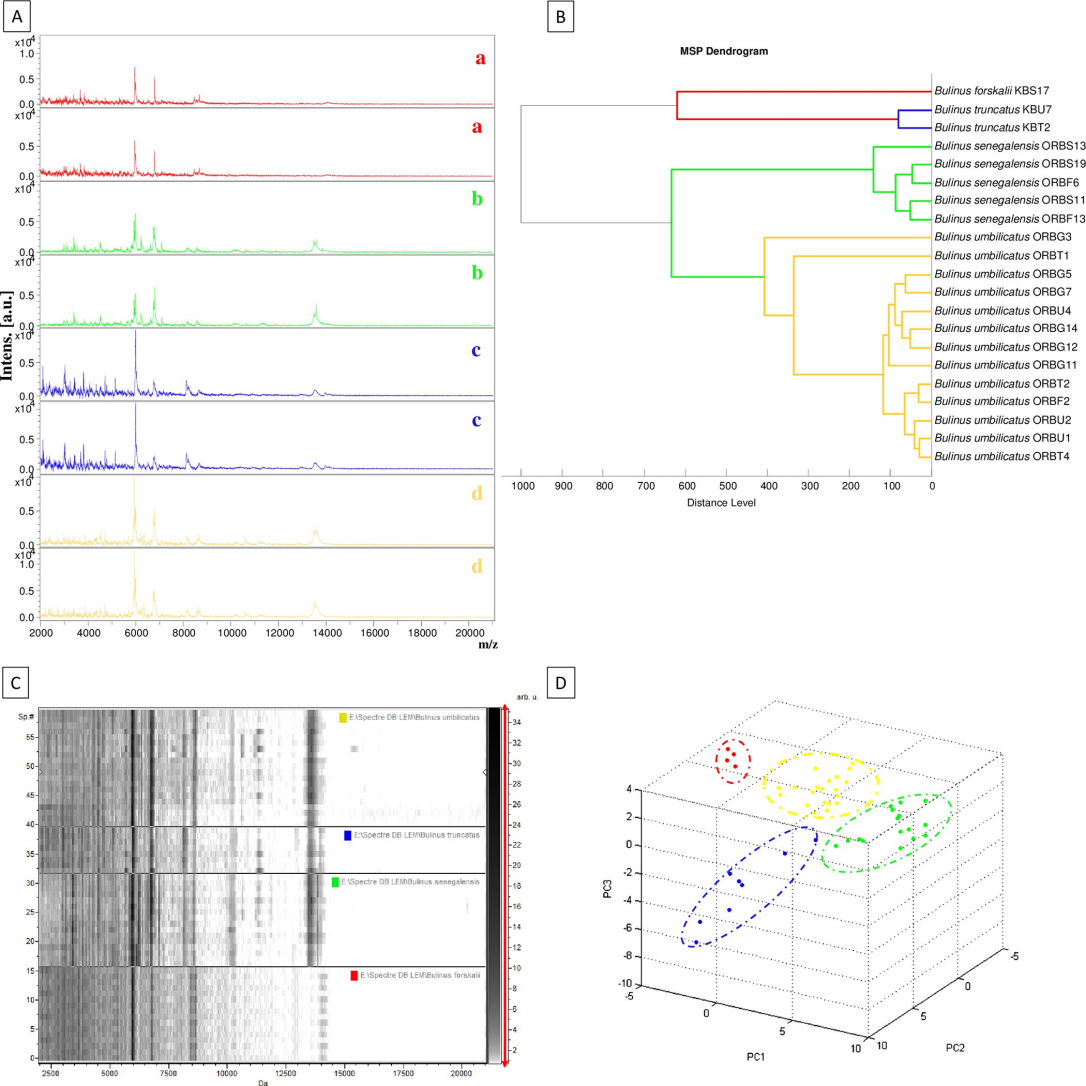
MALDI-TOF MS spectra obtained from different snail species: **[A]** Spectral alignment of four species using flexAnalysis v.3.3 software, (a) *B*. *forskalii*, (b) *B*. *senegalensis*, (c) *B*. *truncatus*, and (d) *B*. *umbilicatus*; **[B]** MS dendrogram created from the MS spectra of the four species using Biotyper v.3.0 software; **[C]** Comparison of the MALDI-TOF MS spectra of the four species by principal component analysis using ClinProTools 2.2 software; **[D]** graphical representation showing the classification of LSVs according to the species present.

Based on the dendrogram (see Fig 4) which showed that *Bulinus* snails clustered hierarchically into four specific clusters, two to four specimens of each morphologically identified *Bulinus* species were randomly selected for DNA-based identification.

Morphological identification (ID): Species determined based on morphological key identification. GB, GenBank.

n: number of snail specimens that were sequenced.

NA: not available, because this region was not sequenced.

**: best identification score obtained with sequencing.

***: GenBank accession numbers of the nucleotide sequences with the highest percentage of identity.

### MALDI-TOF MS identification

A total of 231 snail feet were tested by MALDI-TOF MS, of which only 66 (28.6%) yielded good quality spectra (Fig 4A). MALDI-TOF MS-based identification was considered correct when there was a perfect agreement between the morphological and/or molecular identification. For the update of our laboratory spectral database, 12 good quality spectra were added: *B*. *senegalensis* (n = 4), *B*. *truncatus* (n = 2), *B*. *umbilicatus* (n = 5), and *B*. *forskalii* (n = 1). The rest of the spectra from other specimens (n = 54) were used for the blind test against the updated database (Table 6). All poor quality spectra (n = 165) were excluded from the analysis.

**Table 6:** MALDI-TOF MS-based identification of *Bulinus* snail species from the lake zones of Kankossa and Oued Rawdha, Assaba region, southern Mauritania.

Morphological ID	Number of snail specimens (n)						LSV range	Mean LSV (± SD)
	Total n	Good quality MS spectra	Molecular ID (GB ID %)	MS reference spectra created	Blind test	MS blind test (number of spectra)		
*B*. *senegalensis*	70	11	*B*. *senegalensis* (99.8%) (2)	2	8	*B*. *senegalensis* (6)	2.13–2.48	2.25 ± 0.13
			*B*. *forskalii* (100%) (1)	1		*B*. *forskalii* (2)	1.96–2.10	2.03 ± 0.09
*B*. *forskalii*	56	16	*B*. *senegalensis* (99.8%) (2)	2	13	*B*. *senegalensis* (12)	2.11–2.45	2.32 ± 0.09
			*B*. *umbilicatus* (100%) (1)	1		*B*. *umbilicatus* (1)	2.30	–
*B*. *umbilicatus*	40	16	*B*. *umbilicatus* (100%) (1)	1	14	*B*. *umbilicatus* (7)	2.07–2.43	2.25 ± 0.12
			*B*. *truncatus* (100%) (1)	1		*B*. *truncatus* (7)	1.73–2.22	2.05 ± 0.19
*B*. *truncatus*	32	11	*B*. *umbilicatus* (100%) (1)	1	9	*B*. *umbilicatus* (2)	2.21–2.74	2.48 ± 0.37
			*B*. *truncatus* (100%) (1)	1		*B*. *truncatus* (2)	1.71–2.07	1.89 ± 0.25
*B*. *globosus*	33	12	*B*. *umbilicatus* (100%) (2)	2	10	*B*. *umbilicatus* (10)	2.02–2.43	2.21 ± 0.12
Total	231	66		12	54	(49)		

ID, identification; n, number of snail specimens; MS, mass spectrometry; GB, GenBank; LSV, log score value; SD, standard deviation; ND, not determined. Of the total number of snails tested (second column), the number of specimens with good quality spectra is given in the third column. Among the specimens with good quality spectra, those with sequencing data deposited in GenBank are shown in the fourth column. GenBank ID % in the fourth column refers to the % similarity with the reference sequence deposited in GenBank. The number of snail specimens identified to the species level by PCR sequencing and good quality MS spectra used to create reference MS spectra are given in the fifth column. The remaining samples were tested in a blind test (sixth column). The seventh to ninth columns show the number of *Bulinus* species correctly identified by PCR and MALDI-TOF MS (i.e. with perfect agreement, with the exception of five specimens morphologically identified as *B*. *truncatus* which were not identified by MALDI-TOF MS) and the corresponding LSV. The range of LSV is from 0 to 3. Only good quality spectra were included.

A total of 49 of 54 specimens (90.7%) were successfully identified when tested blinded against the updated database, with LSV ranging from 1.71 to 2.74 (Table 5). All specimens were correctly identified to the species level with the correct identification rate ranging from 22.2% to 100%. Of 70 specimens morphologically identified as *B*. *senegalensis*, 11 yielded spectra of good quality, and two were identified as *B*. *forskalii* in the blind test by MALDI-TOF MS. For snails that were initially identified as *B*. *forskalii* (total number of specimens tested by MALDI-TOF = 56) and *B*. *umbilicatus* (total number = 40) using morphological criteria, 16 of each species yielded spectral profiles of good quality. Molecular methods showed that *B*. *forskalii* snails (according to morphological criteria) were actually *B*. *senegalensis* or *B*. *umbilicatus*. In the blind test, all 12 PCR-confirmed *B*. *senegalensis* snails and one PCR-confirmed *B*. *umbilicatus* snail were correctly identified by MALDI-TOF MS. Likewise, among the snails initially identified as *B*. *umbilicatus* using morphological criteria, some were actually *B*. *truncatus* by PCR. Among these snails, the blind test based on PCR-confirmed *B*. *umbilicatus* (n = 7) and *B*. *truncatus* (n = 7) showed that the MALDI-TOF MS profiles matched with the correct species. Regarding *B*. *truncatus* (total number of specimens identified morphologically and tested by PCR and MALDI-TOF MS = 32), 11 yielded high quality MS spectra. PCR identified these snails as either *B*. *umbilicatus* or *B*. *truncatus*. MALDI-TOF performed poorly for this subset of snails; only two specimens were correctly identified as *B*. *umbilicatus* or *B*. *truncatus*, whereas the other *Bulinus* spp. snails were not identified by MALDI-TOF. All specimens morphologically identified as *B*. *globosus* (33 specimens tested) were actually *B*. *umbilicatus* by PCR. In the blind test of these PCR-confirmed *B*. *umbilicatus* (wrongly identified morphologically as *B*. *globosus*), 12 of 33 snails yielded with good quality spectra. In the blind test, all PCR-confirmed *B*. *umbilicatus* initially identified as *B*. *globosus* using morphological criteria were also correctly identified as *B*. *umbilicatus* using MALDI-TOF MS (Table 6). After Sanger sequencing and MALDI-TOF MS analysis, *B*. *truncatus* and *B*. *forskalii* were the only *Bulinus* spp. found in Kankossa. In Oued Rawdha, only *B*. *senegalensis* and *B*. *umbilicatus* were found among the collected snails.

Representative MS spectra of five snail species identified in the present study were specific with high signal intensities (Fig 4A) and were also observed with the gel view (Fig 4C). The dendrogram made with the spectra (Fig 4B) revealed an inter-species specificity represented by the grouping on distinct branches of specimens of different species (Fig 4D). The spectral data from a single specimen of *B*. *forskalii*, two specimens of *B*. *truncatus*, five *B*. *senegalensis*, and 13 *B*. *umbilicatus* showed that *B*. *truncatus* and *B*. *forskalii* are more closely related than with *B*. *senegalensis*. This result may be due to the geographic origin of the snails. *Bulinus truncatus* and *B*. *forskalii* were the only *Bulinus* spp. found in Kankossa lake, whereas *B*. *senegalensis* and *B*. *umbilicatus*, but not the two *Bulinus* spp. collected in Kankossa, were found in the village of Oued Rawdha.

### Screening of snail infested with *S*. *haematobium* complex parasites

Molecular screening using *Dra*I RT-PCR targeting *Schistosoma* spp. in snails was carried out in 66 snail specimens identified by MALDI-TOF MS. Results showed the presence of *Schistosoma* spp. in 8 (12.1%) of them, including six (five *B*. *senegalensis* and one *B*. *umbilicatus*) from Oued Rawdha and two (one *B*. *truncatus* and one *B*. *forskalii*) from Kankossa. The Ct values ranged from 19.04 to 34.46 (< 35) with a mean (± SD) of 27.95 (± 6.09).

## Discussion

The findings of the present study showed that schistosomiasis poses a serious public health concern in Kankossa and Oued Rawdha villages in Assaba region, southern Mauritania. School children-based surveys of urogenital schistosomiasis in Mauritania have mainly focused on the SRB, classified as a priority zone of intervention, with the prevalence in the lower, middle, or high valleys of the SRB ranging from 17.8–57.4%, 1.3–32.5%, and 25.1–34.3%, respectively [26,29–32]. The prevalence varied considerably, from low (i.e. < 10% of infected schoolchildren) to moderate (10–49% of infected schoolchildren) or even high (≥ 50%) prevalence, according to the now outdated 2002 WHO classification [84]. The present work is the first epidemiological study of urogenital schistosomiasis in Assaba region. The prevalence rates among schoolchildren in this region corresponded to “moderate” prevalence [84]. The relatively high prevalence of urogenital schistosomiasis found in Kankossa could be due to a high level of exposure of the schoolchildren to the contaminated water in the lake which persists throughout the year, while the lower prevalence in Oued Rawdha village could be due to seasonal ponds that depend on rainfall intensity.

The present study showed a moderate prevalence rate (33%) of urogenital schistosomiasis among children aged 6–8 years, compared to the other age groups (9–11 [22.5%] and 12–14 years [17.7%]). There was a statistically significant decrease in the prevalence of urogenital schistosomiasis with increasing age. This observation is in agreement with the hypothesis that increasing age is associated with decreasing prevalence of *S*. *haematobium* infection in the general population due to the development of age-related acquired immunity [85–87]. However, other recent studies performed along the SRB found contrasting results. In other African countries, an association between age and *S*. *haematobium* prevalence has not been demonstrated consistently [88]. Data from other studies also do not seem to support the development of age-associated acquired immunity during childhood and adolescence [44,89–91]. A recent meta-analysis of urogenital schistosomiasis in children reported that age was an associated factor in only four of 15 included studies [17]. Although studies conducted in Africa generally show that school-age children between 5 and 18 years old are the most affected population group, specific contexts related to occupational hazards (e.g., agriculture, mining, laundry, washing or bathing farm animals, crossing contaminated water bodies) may also lead adults and/or females to be at risk of being exposed to *S*. *haematobium*. In Muslim countries, water contact during the obligatory ablutions before prayer has also been reported to be a potential source of infection in all ages and in both sexes although adults tended to have a higher number of water contact through this religious practice [92]. The contradictory data on the most vulnerable age group with relation to urogenital schistosomiasis in Africa call for further large-scale field studies based on similar study design and epidemiological context, including social studies, and meta-analysis for a better understanding of schistosomiasis epidemiology.

Regarding the sex of infected children, our study revealed a higher prevalence of schistosomiasis among boys. This finding is not surprising, particularly in an Islamic country, like Mauritania, where girls are socially precluded from bathing or swimming in open water source while boys are usually allowed to play outside in open water bodies, exposing themselves to the risk of water-borne infections. Similar findings have been reported from some other predominantly Islamic countries in Africa [44,85,93]. However, other studies conducted in Yemen and Sudan reported a similar prevalence rate between the sexes due to the fact that girls are also involved in some water-related domestic activities, such as fetching water and washing clothes and utensils at the contaminated water sources and thus have similar exposure to schistosomiasis as boys [94,95]. Among children, recreational bathing and swimming in *Schistosoma*-infested water are probably the most common mode of transmission; however, infection may occur away from the contaminated water body if cercariae-infested water is transported for house work, bathing, laundry, bathing farm animals, and possibly drinking [96].

Approximately 41% (46% in Kankossa and 29% in Oued Rawdha) of the school-aged children with a positive parasitological test presented with heavy *S*. *haematobium* infections, suggesting that these children have been continually reinfected through regular contact with schistosome-infested freshwater and may run the risk of developing a chronic disease. There were neither intra- nor inter-village differences in the intensity of infection according to the age, sex, and season. This result may possibly be due to the relatively small sample size or the lack of longitudinal data from our two study sites. Kankossa Lake area is characterized by perennial *S*. *haematobium* transmission due to the permanent presence of the water body where children living around the lake are expected to be not only continually infected by *S*. *haematobium* but are also rapidly and frequently reinfected. From an epidemiological viewpoint, the transmission feature in Kankossa Lake is akin to that of SRB [97]. By contrast, in Oued Rawdha, the pond dries up and remains dry for months, during which *S*. *haematobium* transmission is naturally interrupted. This latter type of seasonal transmission zone is characterized by a slower reinfection rate, as shown in other studies [98].

Five *Bulinus* species were identified based on morphological criteria in the present study. However, molecular biology and MALDI-TOF MS techniques revealed only four species, namely *B*. *senegalensis*, *B*. *forskalii*, *B*. *truncatus*, and *B*. *umbilicatus*. Indeed, some specimens initially identified as *B*. *globosus* based on morphology turned out to be *B*. *umbilicatus* after molecular and proteomic analyses. This demonstrates the great interest of MALDI-TOF MS in snail species identification because this technique is less prone to errors than conventional morphology-based identification and malacological expertise is not required to interpret the MS spectra once the reference spectra are stored in the database. Although the initial investment to acquire the instrument is high, compared to a thermal cycler, MALDI-TOF MS is more rapid (a few minutes to obtain the spectral profile and compare it with the reference spectra in the database), requires much less expensive reagents which are, in addition, common to any biological samples, amenable to high throughput analysis, generally requires a few simple steps for sample preparation, does not require a confirmatory analysis of the results, such as sequencing, and does not require a highly skilled technician to operate the instrument [99–101].

MALDI-TOF MS was assessed for the first time to identify several species of freshwater snails belonging to *Bulinus* spp. and *Biomphalaria* spp. a few years ago [58]. In that study, hundreds of frozen snails and specimens that were preserved in ethanol were correctly identified by morphological keys, DNA sequencing (*COI* gene), and MALDI-TOF MS, paving the way for a novel, alternative, rapid, and reliable proteomic approach for snail identification. The reliability of MALDI-TOF to specifically identify *Bulinus forskalii* specimens collected in the field in Senegal was further demonstrated in follow-up studies [58,102]. Further refinement and development of MALDI-TOF protocol involving an addition of spectral profiles of freshwater snail populations occurring in different geographical areas in Senegal to the database have led to the correct identification of the geographic origins of the snails, suggesting that MALDI-TOF MS may even outperform DNA-based technology to identify freshwater snails to the strain level and pinpoint their geographic origin [103]. These recent studies on the application of MALDI-TOF MS in malacology, as well as our present study, suggest that this proteomic technology holds promise for freshwater snail identification, but it still requires further confirmatory studies conducted by other research groups, both in the laboratory and in the field, before its widespread application, as it is already the case in the field of microbiology in developed countries worldwide [99–101].

The malacological surveys carried out during the wet and dry seasons highlighted the evidence, for the first time, that four species of *Bulinus* snails can be potential intermediate hosts of urogenital schistosomiasis in the lake zones in southern Mauritania. The *Bulinus* species collected were *B*. *truncatus*, *B*. *senegalensis*, *B*. *forskalii*, and *B*. *umbilicatus*. The presence of these four species of *Bulinus* had been noted in previous malacological investigations conducted in several localities along the Mauritanian side of the river bank of the Senegal River [4,21,23,24,26]. These species of *Bulinus* can differ in their ability to transmit *S*. *haematobium*, thus contributing to the difference in the prevalence of urogenital schistosomiasis in our study areas. In our study, however, active shedding of *Schistosoma* cercariae was not demonstrated, precluding us from further characterizing and identifying the *Schistosoma* species of the shed cercariae by PCR sequencing.

The snails that were collected were stored in 70% ethanol, an organic substance widely used in the field for transporting samples because it is more practical than freezing. However, only a few (28.6%) gave good quality MALDI-TOF MS spectra. This could be due to the long storage time in ethanol, as several previous works have shown that storage in ethanol may hamper accurate MALDI-TOF MS identification [58,81,104]. However, both dendrogram and PCA analyses of the spectra showed that MALDI-TOF MS is able to distinguish between different snail species, even those of similar morphology. This finding is in line with those of recent studies on MALDI-TOF MS freshwater snail species identification [58,61]. Few studies have been conducted to identify snail species using MALDI-TOF MS. The available data suggest that freezing snails at -20°C or, if possible at -80°C, would preserve the proteins better than ethanol and ensure optimal performance of MALDI-TOF MS for snail identification [58,103].

DNA-based analysis in the present study revealed a high prevalence of infestation of snails by the *S*. *haematobium* complex (12.1%). In Mauritania, PCR-based screening for *Schistosoma* parasites in intermediate snail hosts has not been previously performed. In southern Mauritania, Gbalégba *et al*. [31] assessed *Schistosoma* spp. snail infestation via the cercarial emission test. They found no infected specimens [31]. In contrast, the present PCR-based study revealed a high prevalence of *Bulinus* snail infestation. Moreover, similar snail infestation rates have been observed in schistosomiasis endemic areas in Senegal, when the same PCR protocol was used for the screening [102]. These results highlight the important role of PCR in both snail species identification and epidemiological studies for an accurate assessment of parasite infestation levels in snails. Nonetheless, the infestation test based on cercarial emission is still necessary to confirm the role that a snail species may play in the transmission of different species of *Schistosoma*.

Several limitations need to be taken into consideration for the interpretation of the findings reported in the present work pertaining to malacology. The use of morphological keys for *Bulinus* snails to species level led to an erroneous identification of *B*. *globosus*, which was corrected by PCR and MALDI-TOF MS. However, MALDI-TOF MS did not perform optimally in our study. This technical difficulty was related essentially to poor preservation of snail specimens and the long delay required to transport them from the field to our collaborating laboratory in France. In Senegal, where MALDI-TOF MS is available in Dakar and many snail specimens were frozen before analysis, the results of MALDI-TOF MS to identify *Bulinus* species corresponded to those of molecular identification [58,61,103]. In the context of the present study, PCR sequencing was the most reliable diagnostic method.

Cercarial emission test was not performed due to logistical problems encountered in the field. This circumstance led us to perform PCR to detect *S*. *haematobium*-infested snails. A relatively high proportion (12.1%) of *Bulinus* snails was found to be infected with *S*. *haematobium*-group parasites, possibly due to the fact that *Dra*I is not species-specific and may have detected non-*S*. *haematobium* parasites (e.g. *S*. *bovis*). Recent studies based on cercariae emission test or *S*. *haematobium*-specific molecular methods in naturally-occurring *Bulinus* spp. have shown that the proportion of *Bulinus* spp. infected with *S*. *haematobium* was at most 14%, usually much lower (i.e., less than 3%) [44,98]. Given the fact that *S*. *haematobium*-specific PCR was not performed, the high proportion of *Bulinus* snails with positive *S*. *haematobium* group seen in the present study needs to be interpreted with caution. In addition to the *Dra*I RT-PCR for diagnosis of the *S*. *haematobium* group, several alternative molecular methods can be used to detect *Schistosoma* spp. Previous studies have utilized conventional PCR and sequencing of the COI and ITS regions to differentiate between pure and hybrid strains [61,102,105,106]. Other recent molecular approaches, such as loop-mediated isothermal amplification (LAMP), have also been employed. LAMP, which amplifies DNA at a constant temperature, reduces the need for expensive equipment and is easily adaptable to field laboratories. Field studies have indicated that LAMP is a promising alternative for the diagnosis of *Schistosoma* spp. in resource-limited settings [107,108].

## Conclusions

The present study shows that urogenital schistosomiasis is moderately prevalent in Kankossa and Oued Rawdha lake zones of the Assaba region, southern Mauritania. The *Bulinus* spp. present in these two sites were different, with *B*. *truncatus* and *B*. *forskalii* in Kankossa, and *B*. *umbilicatus* and *B*. *senegalensis* in Oued Rawdha. PCR suggested that all of these four *Bulinus* spp. snails were infected with *S*. *haematobium* group although it cannot be established whether the parasites were *S*. *haematobium*, *S*. *bovis*, or other *S*. *haematobium* group species. Whereas MALDI-TOF MS can discriminate between different *Bulinus* species that are endemic in the SRB and overcomes many limitations of the conventional morphology-based identification of snails, its performance to identify the snails was not satisfactory because too many specimens yielded poor quality spectra in our study. Further assessment of MALDI-TOF MS for the identification of snails collected in the field is warranted, notably by using an alternative preservation system to ethanol. Our study area has not benefited from schistosomiasis control programme. Based on our findings, as well as on the latest updated WHO recommendations, we advocate for the implementation of appropriate schistosomiasis control strategies, including an annual single-dose preventive therapy with PZQ in all residents in Kankossa and Oued Rawdha (with the exception of young children < 2 years old and pregnant women during the first trimester of pregnancy), and increased community awareness.

## Supporting information

S1 Table Primers used in the PCR reactions for the identification of *Bulinus* snails collected in Kankossa and Oued Rawdha villages, Assaba region, southern Mauritania. (DOCX)

S1 Appendix R packages and functions used for statistical analyses. (DOCX)

S2 Appendix COI and 28S rRNA sequences and GenBank accession numbers of *Bulinus* spp. collected in Kankossa and Oued Rawdha. (DOCX)

S1 Data Data source for age and sex of all included school children in Kankossa and Oued Rawdha (n = 450). (XLSX)

S2 Data Data source for the prevalence and intensity of urogenital schistosomiasis with respect to age, sex, and season (presented in Fig 2). (XLSX)
