# Supplementary material for: Urogenital schistosomiasis in schoolchildren in the lake zones of Kankossa and Oued Rawdha, southern Mauritania: The first parasitological and malacological survey
Source: PLoS Negl Trop Dis. 2024 Sep 25;18(9):e0012505. doi: 10.1371/journal.pntd.0012505 (PMC11458011; doi:10.1371/journal.pntd.0012505)
Supplement: S1 Table — (DOCX) [file pntd.0012505.s001.docx]

**S1 Table**: Primers and probe used in the PCR amplification for the identification of *Bulinus* snails and *Schistosoma haematobium*-infected snails collected in Kankossa and Oued Rawdha villages, Assaba region, southern Mauritania

| **Genome** | **Gene** | **Primer name** | **Forward/Reverse** | **Primer sequences (5’-3’)** | **Target organism** | **Reference** |
| --- | --- | --- | --- | --- | --- | --- |
| Mitochondria | *COI* | Bglob-CoxF | F | TGGTAGGAACCGGACTTTCA | *Bulinus globosus* | [74] |
|  |  | HC02198 | R | TAAACTTCAGGGTGACCAAAAAATCA |  | [73] |
|  |  | LCO1490 | F | GGTCAACAAATCATAAAGATATTGG | *Bulinus* spp. | [73] |
|  |  | HC02198 | R | TAAACTTCAGGGTGACCAAAAAATCA |  |  |
| Nuclear | 28S rRNA | 28SF4 | F | AGTACCGTGAGGGAAAGTTG | *Bulinus* spp. | [75] |
|  |  | 28SR5 | R | ACGGGACGGGCCGGTGGTGC |  |  |
| Nuclear | *Dra*I | Sh1 | F | GATCTCACCTATCAGACGAAAC | *S. haematobium* group | [78] |
|  |  | Sh2 | R | TCACAACGATACGACCAAC |  |  |
|  |  | Sh-probe | Probe* | TGTTGGTGGAAGTGCCTGTTTCGCAA |  | [79] |

*COI*, mitochondrial cytochrome oxidase 1; F, forward primer; R, reverse primer. The primer pairs Bglob-CoxF and HC02198 are specific for *Bulinus globosus*. The other primer pairs, LCO01490/HC02198 and 28SF4/28SR5, are specific for the genus *Bulinus*. *Dra*I is a 121-bp repetitive sequence. *The fluorescent labelled hydrolysis probe used in RT-PCR.
