# Supplementary material for: Urogenital schistosomiasis in schoolchildren in the lake zones of Kankossa and Oued Rawdha, southern Mauritania: The first parasitological and malacological survey
Source: PLoS Negl Trop Dis. 2024 Sep 25;18(9):e0012505. doi: 10.1371/journal.pntd.0012505 (PMC11458011; doi:10.1371/journal.pntd.0012505)
Supplement: S1 Appendix — (DOCX) [file pntd.0012505.s002.docx]

install.packages("crosstable")

install.packages("dplyr")

install.packages("readxl")

install.packages("MASS")

install.packages("mice")

mydata <- read_excel("G:/Mon Drive/document/BASE.xlsx")

mydata[mydata==""]<-NA

mydata<- as.data.frame(unclass(mydata),stringsAsFactors=TRUE)

summary(mydata)

# We create a list of variables to convert into factor

vars<-c("ID","NIVEAU","SEXE","INTENSITE","INFECTION","AGE")

# We apply the "factor" transformation to all the elements in the "vars" list using the "lapply" function.

mydata[vars]<-lapply(mydata[vars],factor)

summary(mydata)

table1=crosstable(mydata, c(NIVEAU,SEXE,AGE,INTENSITE,INFECTION), showNA="no",test=TRUE,percent_digits=1,total="all",percent_pattern="{n} ({p_col})")

as_flextable(table1, keep_id=FALSE)

table1=crosstable(mydata, c(NIVEAU,SEXE,AGE,INTENSITE),by=INFECTION, showNA="no",test=TRUE,percent_digits=1,total="all",percent_pattern="{n} ({p_row})")

as_flextable(table1, keep_id=FALSE)

#Significant factors associated with Infection: multivariate logistic regression

model <- glm(INFECTION ~ SEXE + AGE + NIVEAU ,data = mydata, family = binomial)

summary(model)

#Odds ratios of variables

exp(coef(model))

exp(confint(model))
