# Supplementary material for: Urogenital schistosomiasis in schoolchildren in the lake zones of Kankossa and Oued Rawdha, southern Mauritania: The first parasitological and malacological survey
Source: PLoS Negl Trop Dis. 2024 Sep 25;18(9):e0012505. doi: 10.1371/journal.pntd.0012505 (PMC11458011; doi:10.1371/journal.pntd.0012505)
Supplement: S2 Appendix — (DOCX) [file pntd.0012505.s003.docx]

------------------------------------------------------- COI ----------------------------------------------------

>Seq1_COI_gene_Kankoussa_KBU7_*Bulinus truncatus*_Mauritania_GenBank accession no. OR921236_459 bp

CTTCTGTTCTAATAGATGAACATTTTTATAATGTTATTGTTACTGCACATGCTTTTATTATAATTTTTTTTATAGTTAAACCTATAATAATTGGAGGATTTGGGAATTGAATAGTTCCTCTTCTTATTGGTGCTCCTGATATAAGATTCCCTCGTATAAATAATATATCATTTTGACTTTTACCACCATCTTTTATTTTATTATTGGTTTCTTCTATAGTCGAAGGAGGGGTTGGAACAGGATGAACTGTATATCCTCCTTTAAGAGGACCTATTGCACATGGAGGTGCTTCAGTTGATTTAGCTATTTTTTCGCTTCATTTAGCTGGGATATCATCAATTTTAGGGGCAATTAATTTTATTACTACAATTTTCAATATACGGGCACCAGGAATTACTATAGAACGATTATCTTTATTTGTATGATCTGTACTAATTACTGCTTTTTTATTACTTTTAT

>Seq2_COI_gene_Kankoussa_KBT2_*Bulinus truncatus*_Mauritania_GenBank accession no. OR921237_459 bp

CTTCTGTTTTAATAGATGAACATTTTTATAATGTTATTGTTACTGCACATGCTTTTATTATAATTTTTTTTATAGTTATACCTATAATAATTGGAGGATTTGGGAATTGAATAGTTCCTCTTCTTATTGGTGCTCCTGATATAAGATTTCCTCGTATAAATAATATATCATTTTGACTTTTACCACCATCTTTTATTTTATTATTGGTTTCTTCTATAGTCGAAGGAGGAGTTGGAACAGGATGAACTGTATATCCTCCTTTAAGAGGACCTATTGCACATGGAGGTGCTTCAGTTGATTTAGCTATTTTTTCACTTCATTTAGCTGGGATATCATCAATTTTAGGAGCAATTAATTTTATTACTACAATTTTCAATATACGGGCACCAGGAGTTACTATAGAACGATTATCTTTATTTG

TATGATCTGTACTAATTACTGCTTTTTTATTACTTTTAT

>Seq3_COI_gene_Oued Rawdha_ORBT2_*Bulinus umbilicatus*_Mauritania_GenBank accession no. OR921238_459 bp

CTTCTGTTCTCATAGATGAACATTTTTATAATGTAATTGTTACAGCACATGCTTTTATTATGATTTTTTTTATGGTTATACCAATAATAATTGGTGGTTTTGGGAATTGAATAGTTCCTTTATTAATTGGGGCACCTGATATAAGATTCACACGAATGAATAATATATCGTTTTGATTACTTCCTCCTTCATTTATTTTATTGTTAGTATCTTCAATAGTAGAAGGTGGAGTAGGAACAGGATGAACTGTTTACCCTCCACTAAGAGGGCCTATTGCTCATGGGGGTGCTTCAGTTGATTTAGCTATTTTTTCTCTACATTTAGCTGGTATATCTTCTATTTTAGGTGCTATTAATTTCATTACAACAATTTTTAATATGCGGGCTCCTGGAATTACAATAGAGCGACTATCATTATTTGTTTGATCTGTTTTAATTACTGCTTTTTTACTTCTTTTAT

>Seq4_COI_gene_Oued Rawdha_ORBG7_*Bulinus umbilicatus*_Mauritania_GenBank accession no. OR921239_459 bp

CTTCTGTTCTCATAGATGAACATTTTTATAATGTAATTGTTACAGCACATGCTTTTATTATGATTTTTTTTATGGTTATACCAATAATAATTGGTGGTTTTGGGAATTGAATAGTTCCTTTATTAATTGGGGCACCTGATATAAGATTCCCACGAATGAATAATATATCGTTTTGATTACTTCCTCCTTCATTTATTTTATTGTTAGTATCTTCAATAGTAGAAGGTGGAGTAGGAACAGGATGAACTGTTTACCCTCCACTAAGAGGGCCTATTGCTCATGGGGGTGCTTCAGTTGATTTAGCTATTTTTTCTCTACATTTAGCTGGTATATCTTCTATTTTAGGTGCTATTAATTTCATTACTACAATTTTTAATATGCGGGCTCCTGGAATTACAATGGAGCGACTATCATTATTTGTTTGATCTGTTTTAATTACTGCTTTTTTACTTCTTTTAT

----------------------------------------------------- 28S rRNA ---------------------------------------------------

>Seq1_28Sgene_Kankoussa_KBU7_*Bulinus truncatus*_Mauritania_GenBank accession no.: OR921249_538 bp

CCACCTGGACCGCTCGGAGAACGGTCCTCGCTTTCACTGCGCCTTTGGGTTTCGTACCGCCCAATGACTCGCGCGCATGTTAGACTCCTTGGTCCGTGTTTCAAGACGGGTCGGGTGGTAGGCCGACAGATTCGCCACTGACCCCGTATCTCACCCGGCAGAACCCGATCCGACGCAGAGGCGCGGTCGGCGGGCACGGCAGAAACCAGTCCCCACCAGTCGAACGCCCCCGCGAGGCCGGGCAGCCTCAAGGCGAAAGGTCGATCCTCGGTCCCCGAACGGCTCGCCGCGGGCAGCCCGCGACACGGCCCAGAGGACCGCGCCGCGCAAAGCCAGCCCGAGCTTATCGCCGCCCAGAAACCGATCGTAGCGCTCCGCCCGCAGAAAGTGCACGCGGCGTGACCCGGGTCGTCACCGCCGGAGGGTCCGAGGGATCCCAAGCCGGCGGCAGCCCCGAGCCGGACGCCGCGCTGAATTCCGCGGGCCGACTTTTGCGGAACCACCCGTTTACCTCTGAGCGGTTTCACGTACTCTTGAA

>Seq2_28Sgene_Kankoussa_KBT2_*Bulinus truncatus*_Mauritania_GenBank accession no. OR921250_538 bp

CCACCTGGACCGCTCGGAGAACGGTCCTCGCTTTCACTGCGCCTTTGGGTTTCGTACCGCCCAATGACTCGCGCGCATGTTAGACTCCTTGGTCCGTGTTTCAAGACGGGTCGGGTGGTAGGCCGACAGATTCGCCACTGACCCCGTATCTCACCCGGCAGAACCCGATCCGACGCAGAGGCGCGGTCGGCGGGCACGGCAGAAACCAGTCCCCACCAGTCGAACGCCCCCGCGAGGCCGGGCAGCCTCAAGGCGAAAGGTCGATCCTCGGTCCCCGAACGGCTCGCCGCGGGCAGCCCGCGACACGGCCCAGAGGACCGCGCCGCGCAAAGCCAGCCCGAGCTTATCGCCGCCCAGAAACCGATCGTAGCGCTCCGCCCGCAGAAAGTGCACGCGGCGTGACCCGGGTCGTCACCGCCGGAGGGTCCGAGGGATCCCAAGCCGGCGGCAGCCCCGAGCCGGACGCCGCGCTGAATTCCGCGGGCCGACTTTTGCGGAACCACCCGTTTACCTCTGAGCGGTTTCACGTACTCTTGAA

>Seq3_28Sgene_Oued Rawdha_ORBT2_*Bulinus umbilicatus*_Mauritania_GenBank accession no. OR921251_534 bp

CCACCTGGGCCGCTCGGAGAACGGTCCTCGCTTTCACTGCGCCTTTGGGTTTCGTACTACCCAATGACTCGCGCGCATGTTAGACTCCTTGGTCCGTGTTTCAAGACGGGTCGGGTGGTAGGCCGACAGATTCGCCACTGACCCCGGATCTCGCCCGGCAGACCCGATCCGACGCAGAGGCGCGGTCGGCGGGCACGGCATGAAGCCAGTCCCCACCAGTCGAACGCCCCCGCGAGGCCGGGAAGCCACGAAGCGAAAGGTCGATCCTCGGTCCCCAGGCGGCTCGCCGCGGCAGCCCGCGACACGGCCCTGGAGCCGCGCCGCGCAAAGCCACCGGGCTTGTCGCCGCCCGAAAACCGATCGTAGCGCTCCGCCCGCAGAAAGTGCACGCGGCGTCGCCCGGCAGAAGCCGCCGCCGGAGGGTCCGAGGGATCCCTGCCGGCAGCGACGCCAGGCTACCGCCGCGCTGAATTCCGCGGGCCGACTTTTGCGGAACCACCCGTTTACCTCTGAGCGGTTTCACGTACTCTTGAA

>Seq4_28Sgene_Oued Rawdha_ORBG7_*Bulinus umbilicatus*_Mauritania_GenBank accession no. OR921252_531 bp

CCACCTGGGCCGCTCGGAGAACGGTCCTCGCTTTCACTGCGCCTTTGGGTTTCGTACTACCCAATGACTCGCGCGCATGTTAGACTCCTTGGTCCGTGTTTCAAGACGGGTCGGGTGGTAGGCCGACAGATTCGCCACTGACCCCGGATCTCGCCCGGCAGACCCGATCCGACGCAGAGGCGCGGTCGGCGGGCACGGCATGAAGCCAGTCCCCACCAGTCGAACGCCCCCGCGAGGCCGGGAAGCCACGAAGCGAAAGGTCGATCCTCGGTCCCCAGGCGGCTCGCCGCGGCAGCCCGCGACACGGCCCTAGAGCCGCGCCGCGCAAAGCCACCGGGCTTGTCGCCGCCCGAAAACCGATCGTAGCGCTCCGCCCGCAGAAAGTGCACGCGGCGTCGCCCGGCAGAAGCCGCCGGAGGGTCCGAGGGATCCCTGCCGGCAGCGACGCCAGGCTACCGCCGCGCTGAATTCCGCGGGCCGACTTTTGCGGAACCACCCGTTTACCTCTGAGCGGTTTCACGTACTCTTGAA

>Seq5_28Sgene_Oued Rawdha_ORBS11_*Bulinus senegalensis*_Mauritania_GenBank accession no. OR921253_539 bp

CCACCTGGACCGCTCGGAGAACGGTCCTCGCTTTCACTTCGCCTTTGGGTTTCGTACAGCCCAGTGACTCGCGCGCATGTTAGACTCCTTGGTCCGTGTTTCAAGACGGGTCGGGTGGTGGGCCGACAGATTCGCCACCGACCCCGGATGCTCGCCCGGCTGGCCCCGGTCCAGCGCAGGGATGCGGTCGGCGGGCACGGCCAGAAGCCAGTCCCCGCCAGTCGAACACCCCCGCGAGGCCGGGCAGCCTCGAAGCGAAAGGTCGATCCTCGGTCCCCAAGCAGCTCGCCAGCAGGCAGCCCGAGACACGGCCCGTAGACCGCGCCCCGCAAAGCCGCCCGGGCTTGTCGCCGCCCGAAAACCGATCGTGGCGCTCAGCCCGCAGAAAGTGCACGCGGCATCACCCGGGTCGACACCACCGGAGGGTCCGAGGGATCCCAAACCGGCGGCAGCCCCAGGCAGGACGCCGCGCTGAATTCCGCGGGCCGACTTTTGCGGAACCACCCGTTTACCTCTGAGCGGTTTCACGTACTCTTGAA
